# An Improved Method to Extract DNA from 1 ml of Uncultured Amniotic Fluid from Patients at Less than 16 Weeks’ Gestation

**DOI:** 10.1371/journal.pone.0059956

**Published:** 2013-04-02

**Authors:** Anne-Laure Mosca-Boidron, Laurence Faivre, Serge Aho, Nathalie Marle, Caroline Truntzer, Thierry Rousseau, Clémence Ragon, Muriel Payet, Christelle Thauvin-Robinet, Julien Thevenon, Salima El Chehadeh, Fréderic Huet, Paul Sagot, Francine Mugneret, Patrick Callier

**Affiliations:** 1 Département de Génétique, Hôpital Le Bocage, Dijon, France; 2 Service d’Epidémiologie et d’Hygiène Hospitalière, Hôpital Le Bocage, Dijon, France; 3 Plateforme Protéomique – CLIPP, Hôpital Le Bocage, Dijon, France; 4 Service de Gynécologie, Hôpital Le Bocage, Dijon, France; 5 Pédiatrie I, Hôpital d’enfants, Dijon, France; Tor Vergata University of Rome, Italy

## Abstract

The aim of this study was to develop an improved technique for DNA extraction from 1 ml of uncultured AF from patients with a gestational age less than 16 weeks and to allow the use of array-CGH without DNA amplification. The DNA extraction protocol was tested in a series of 90 samples including 41 of uncultured AF at less than 16 weeks of gestation. Statistical analyses were performed using linear regression. To evaluate the sensitivity and the specificity of array-CGH on 1 ml of uncultured AF, five samples with an abnormal karyotype (three with aneuploidy, two with structural abnormalities) and five with a normal karyotype were studied. This protocol was reproducible and we were able to show a great improvement with higher yield of DNA obtained from all patients, including those with a gestational age less than 16 weeks (p = 0.003). All chromosomal abnormalities were detected and characterized by array-CGH and normal samples showed normal profiles. This new DNA extraction protocol associated with array-CGH analysis could be used in prenatal testing even when gestational age is less than 16 weeks, especially in cases with abnormal ultrasound findings.

## Introduction

The majority of unbalanced chromosomal rearrangements diagnosed prenatally have been identified by prenatal karyotyping and the prevalence was estimated at 4% by the French Biomedicine Agency (http://www.agence-biomedecine.fr). Prenatal karyotyping is limited by resolution (10 Mb), requires cell culture and results are generally available within 2 weeks. In an attempt to overcome these limitations, alternative detection methods based on comparative genomic hybridization microarray (array-CGH) have been applied to prenatal diagnoses using higher resolution microarray [1]–[16].

Historically, amniotic fluid (AF) samples were not considered for use in array-CGH analyses because of insufficient quantities of DNA. In prenatal diagnosis, it was shown that fetal cells extracted from amniotic fluid could be analyzed by array-CGH. Fetal DNA is present in large quantities in amniotic fluid, and Rebello et al. [17] showed that it could be extracted and analyzed by PCR from uncultured amniocytes. Bianchi et al. [18] showed that fetal DNA could be extracted from amniotic fluid supernatant (AF cffDNA) and used for genetic research and clinical applications. It has since been discovered that with an appropriate protocol, it is possible to release microgram amounts of DNA from amniotic fluid and perform array-CGH analysis [8]. The minimum volume of uncultured amniotic fluid was 1 to 4 ml with or without Whole Genome Amplification (WGA) [2], [3], [7]–[9]. However, in many publications, WGA was used because it has been shown to amplify the original DNA when not enough DNA is available [3], [7]–[9], [14]. Cell culture was not required in some of these published methods [1], [2], [4], [5]. However, fetal DNA was reliably extracted from cell-free fetal DNA [5] or uncultured amniotic fluid (AF) [2], [3], [8], [15] and DNA yields have been reported to be influenced by gestational ages [8].

Here, we describe an improved technique for DNA extraction from 1 ml of uncultured amniotic fluid from patients with a gestational age less than 16 weeks. The technique allows array-CGH analysis to detect chromosomal copy number imbalances prenatally.

## Materials and Methods

### DNA Extraction Protocol

In the first step, we developed a new DNA extraction protocol, which was tested on 90 samples of uncultured amniotic fluid from a bank of 1400 samples of amniotic fluid collected in our laboratory since 2002 (average of 1 ml) and stored at −80°C. The indications for testing included advanced maternal age, abnormal ultrasound findings, a history of chromosomal abnormalities and convenience. Among the ninety samples of uncultured amniotic fluid (gestation age ranging from 13.1 to 34 weeks), 41 samples showed a gestational age of less than 16 weeks. Among these, five samples showed chromosomal abnormalities identified by conventional karyotype or FISH. We tested the previously published protocols to isolate genomic DNA from uncultured amniocytes using 1 ml of AF [2], [3]. We did not manage to reproduce the results for DNA extraction from uncultured amniotic fluid using the Rickman et al. [2] protocol. We performed new DNA extraction from 1 ml of uncultured amniocytes with the QIAmp DNA Mini kit (Qiagen, Valencia, CA), but with several modifications [8]. Indeed, we used smaller volumes of lysis buffer, wash buffer and elution buffer without RNase. We tested nine cases <16 WG with Rnase treatment. The DNA was purified and concentrated using a DNA Clean Concentrator kit (Zymo Research, CA). Briefly, the amniotic fluid was centrifuged for 15 min at 4000 rpm at room temperature. The supernatant was removed and the pellet retained. The pellet was washed in 500 µl 1×PBS by vortexing gently. Once the pellet had been resuspended, 50 µl of proteinase K and 600 µl Buffer AL were added. The tube was placed in a water bath at 58°C for 20 minutes, 300 µl of absolute ethanol at −20°C was added and the solution was mixed. A QIAamp DNA Mini Kit ™ column was placed on a collecting tube and half (∼700 µl) of the solution was transferred. The solution was centrifuged for 2 minutes at 14000 rpm; the collection tube was emptied and the second half of the solution was transferred and centrifuged for 2 minutes at 14000 rpm. The column was placed on a new collection tube and DNA was washed with 500 µl of buffer AW1 and AW2 and 50 µl of AE buffer according to the protocol provided by Qiagen. Purification and Concentration with the DNA Clean & Concentrator ™ Kit were performed according to the protocol provided by Zymo. Approximately 10 µl of purified concentrated DNA was obtained and were estimated using a Nanodrop spectrophotometer (NanoDrop Technologies, Wilmington, DE). The experiments were only performed once in each case, due to the limited quantity of amniotic fluid (1 ml).

### Array-CGH

In the second step, we performed microarray Agilent 4×44 K on 10 samples of uncultured amniotic fluid samples of less than 16 weeks of gestation to evaluate the sensitivity of the protocol for the detection of abnormalities using five chromosomal abnormalities and the specificity using five normal karyotypes (Table 1). Furthermore, 12 samples of uncultured amniotic fluid of more than 16 weeks of gestation were analyzed by array-CGH (Table 2). Array-CGH was performed using the Agilent Human Genome CGH Microarray Kit 44 K (Agilent Technologies, Santa Clara, CA). This platform is a high-resolution 60-mer oligonucleotide-based microarray representing 44,000 probes (44 K) that allow a genome-wide survey and molecular profiling of genomic aberrations with an average resolution of ∼35 kb [19]. Array-CGH experiments were performed with the maximum amount of DNA available. Array-CGH analysis was performed according to the Agilent protocol with minor protocol modifications: DNA was labeled by direct incorporation of Cya-5 and Cya-3 using a CGH labeling kit for Oligo-array (Enzo Life Sciences, COVALAB, France) for 4 hr, and labeled products were purified by QIAamp DNA Mini kit (Qiagen, Valencia, CA). A graphical overview was obtained using CGH analytics software (v4.0), and the statistical algorithms ADM-2 according to a sensitivity threshold at 6.0 and a moving average window of 0.5 Mb [20]. Mapping data were analyzed on the human genome sequence build hg18 using ensemble (www.ensembl.org). Copy Number Variations (CNV) were assessed in the Database of Genomic Variants (http://projects.tcag.ca/variation/).

**Table 1:** Summary of karyotype, array-CGH results, indication and DNA isolated from 1 ml of uncultured amniotic fluid at less than 16 weeks’ gestation.

Normal	Samples	Karyotype	Array-CGH (size)	Indication	Gestation (wks)	DNA yield per 1 ml of AF (ng)	DLRS
Aneuploidy	1	45,X	arr Xx1	Cystic hygroma	14	78	0.26
	2	47,XY,+21	arr 21X3	AMA	15	116.2	0.25
	3	47,XY,+21	arr 21X3	Cystic hygroma	15	50.4	0.31
Structural abormalities	4	46,XY.ish del(5)(p15.3)	arr 5p15.33p15.31 X1 (6.4 Mb)	Convenience	15.1	140	0.09
			arr 5p15.31p15.2 X3 (2.3 Mb)				
	5	46,XY,dup(6)(q22q27)	arr 6q22.2q27 X3 (50 Mb)	Cystic hygroma	15.1	190	0.25
			arr 6q27 X1 (1 Mb)				
Normal	6	46,XX	arr(1-22,X)x2	R>1/100	15	28.3	0.60
	7	46.XX	arr(1-22,X)x2	R>1/100	15	60	0.48
	8	46,XX	arr(1-22,X)x2	R>1/212	15	55.1	0.41
	9	46,XY	arr(1-22)x2, (XY)x1	Cystic hygroma	15	41.5	0.36
	10	46,XX	arr(1-22,X)x2	AMA	15.4	97	0.26

AMA, Advanced maternal age; IUGR, intrauterine growth retardation; AF, amniotic fluid; R, risk evaluation after triple test screening’.

**Table 2:** Summary of karyotype, array-CGH results, indication and DNA isolated from 1 ml of uncultured amniotic fluid with chromosomal rearrangement at more than 16 weeks of gestation.

Chromosomal rearrangement	Samples	Karyotype	Array-CGH (size)	Indication	Gestation (wks)	DNA yield per 1 ml of AF (ng)	DLRS
Aneuploidy	11	47,XX,+18	arr 18X3	Multiple anomalies	21	230	0.29
	12	47,XX,+21	arr 21X3	AMA	20	330	0.28
Structural abormalities	13	47,XX,+der(22)(pterq12)	arr 22q11.21 X3 (3.8 Mb)	AMA	17	395	0.16
	14	46,XY,del(2)(q24q31)^a^	arr 2q24.3q31.1 X1 (10 Mb)	Cystic hygroma	17.7	260	0.18
	15	arr 3p13 X1 (3 Mb)^b^	arr 3p14.1p13 X1 (2.3 Mb)	Ventricular Kyste	33	550	0.19
	16	46,XX,del(7)(q33)	arr 7q33q37 X1 (22 Mb)	IUGR, heart defect	22.8	430	0.17
	17	arr 9q33.3 X1 (3.5 Mb)^b^	arr 9q33.3 X1 (2.7 Mb)	IUGR	25	500	0.16
	18	46,XX,dup(11)(pter-q25::q25-q22.2)	arr 11q22.3q24.3 X3 (24 Mb)	Convenience	20.6	840	0.14
			arr 11q24.3q25 X1 (4 Mb)				
	19	46,XX,r(13)	arr 13q31.2q34 X1 (26 Mb)	IUGR, holoprosencephaly	24	640	0.15
	20	46,XY,dup(17)(p12pter)	arr 17p13.3p13.2 X3 (3.6 Mb)	Multiple anomalies	26	230	0.18
	21	45,XY,der(15;18)(q10;q10)	arr 18p12p10 X1 (14 Mb)	Holoprosencephaly	26.7	570	0.16
	22	46,XY.ish del(22)(q11.2q11.2)	arr 22q11.21 X1 (2.4 Mb)	Heart defect	30	180	0.25

AMA, Advanced maternal age; IUGR, intrauterine growth retardation; AF, amniotic fluid.

Detected by ^a^postnatal karyotype or by ^b^postnatal array-CGH.

### Statistical Method

In order to compare our improved technique with previously published techniques. Data reported by Bi et al. [8] were recaptured in order to compare them with ours, under the assumption that the populations studied shared the same baseline characteristics. Amounts of DNA and gestational age were described using their means, standard deviations, medians, and 95% confidence intervals. The normal distribution of DNA quantities and gestational age was checked using polynomial transformations. The logarithmic transformation was the most appropriate in this task and also stabilized their variances. The relationship between DNA quantities and gestational age was tested using a linear regression model, with a robust estimator of variance. The difference between DNA quantities in our study and those of Bi et al. [8] was assessed using the same model (which in this case corresponds to an analysis of variance). A multiple linear regression model, with a robust estimator variance was used to take into account gestational age and type of study simultaneously (Bi vs. ours), as predictors of DNA quantities. Whatever the model considered, logarithmic transforms were used. The adequacy of these models was tested using residuals analysis and by looking at the coefficient of determination.

## Results

### DNA Extraction

We extracted between 28.3 and 840 ng of DNA per 1 ml of AF from each of the ninety uncultured samples (Table 3). There was a correlation between the duration of gestation and the amount of DNA (which is already known) and statistically significant differences were observed between the Bi et al. study [8] and our study (109.4 ng vs. 218.6 ng, respectively; p = 0.0147 See Table 4). Moreover, the multiple linear regression analysis showed the same results (Table 5).

**Table 3:** Summary of DNA yield, purity (260/280 and 260/230 reading) extract by this optimized protocol of the 90 uncultured amniotic fluid.

Weeks gestational age	DNA yield per 1 ml of AF (ng)	A260/A280ratios	A260/230 ratios	Samples
13.1	90,1	1,59	1,4	
14	188	1,9	1,43	
14	78	2.1	0,74	1
14.7	67	1,58	0,95	
15	116.2	2.5	1	2
15	115.5	1.93	0,71	
15	50.4	2.04	1,15	3
15	76.5	1.83	0,91	
15	132.6	1.44	0,69	
15	238.7	1.75	0,67	
15	66.3	2.66	1,1	
15	76.8	2.53	0,34	
15	55.1	2.55	0,4	8
15	84.7	2.31	0,58	
15	28.3	2.63	0,36	6
15	92.4	2.06	0,63	
15	41.5	1.94	0,27	9
15	69.3	2.28	0,65	
15	60	1,76	0,73	7
15	97	1,68	0,87	
15.1	140,0	1,94	1,31	4
15.1	190,0	1,8	1,12	5
15.3	70,1	1,97	0,71	Rnase
15.3	55,8	2,2	1,08	
15.3	51	3,34	0,66	Rnase
15.4	97,0	1,62	0,9	10
15.4	82,0	1,7	0,75	
15.4	82	1,4	0,66	
15.6	53	1,39	0,82	Rnase
15.6	65	1,19	0,41	Rnase
15.6	53	1,82	0,66	Rnase
15.6	63,7	1,66	0,73	
15.7	61	1,53	1	
15.7	87	1,78	1,19	Rnase
15.7	72,8	2,18	1,22	
16	169,0	1,77	1,09	
16	79,6	1,98	0,46	Rnase
16	94,9	1,5	1,09	Rnase
16	50,6	1,6	0,82	
16	67,7	1,66	0,86	
16	70	1,77	0,97	Rnase
17	395,0	1,86	1,65	13
17	223,0	1,69	1,5	
17.4	113,0	2,03	1,16	
17.7	262,0	1,85	1,8	14
17.7	172,0	1,76	1,01	
18.4	213,0	1,92	1,33	
18.6	108,0	1,56	1,18	
18.8	259,0	1,92	1,48	
19	268,0	1,92	1,65	
19.7	259,0	1,94	1,51	
20	330,0	1,78	1,6	12
20	263,8	1,81	1,55	
20.1	219,5	1,82	1,4	
20.1	89,0	1,31	0,86	
20.3	134,8	1,9	1,51	
20.3	381,6	1,83	1,74	
20.3	208,6	1,84	1,55	
20.3	276,6	1,8	1,83	
20.6	840,0	1,82	2,01	18
20.6	401,1	1,83	1,77	
20.6	232,9	1,77	1,81	
20.7	325,1	1,84	1,77	
20.7	223,8	1,76	1,52	
21	230,0	1,87	1,47	11
21	652,0	1,84	1,97	
21	427,5	1,84	1,97	
21.1	400,4	1,84	1,77	
21.1	250,4	1,96	1,89	
21.6	434,6	1,8	1,97	
21.7	234,5	1,98	1,18	
21.8	278,5	1,73	1,37	
22.1	420,4	1,89	1,94	
22.7	336,2	1,75	1,69	
22.8	430,0	1,79	1,89	16
23	366,0	1,86	2	
24	644,0	1,81	0,37	19
25	505,0	1,86	1,8	17
26	235,0	1,87	1,42	20
26.7	570,0	1,87	1,93	21
28	470,4	1,86	1,95	
29.7	216,0	1,94	1,59	
30	181,0	2,29	1,24	22
30.8	180,0	1,86	1,98	
32	349,0	1,82	1,45	
32	277,0	1,82	1,66	
33	553,0	1,86	1,83	15
33.1	385,0	1,84	1,89	
33.1	415,0	1,91	1,71	
34	452.1	1.88	1,23	

**Table 4:** Comparison of DNA yield and gestational age with Bi et al. [2008] study.

		Average DNA yield (ng/ml)		Number of cases	
		Bi *et al*., 2008	Our study - Mean (95% confidence limits)	Bi *et al*., 2008	Our study
Gestation (wks)	≤16	36.4	87.3 (73.5–101.1)	2	41
	>16–20	120.7	269.5 (204–335)	10	23
	21–22	191.8	372.2 (288.4–456.1)	3	11
	23–34	no data	386.5 (304.9–468.3)	no data	15
	Total	109.4	218.6	15	90

**Table 5:** Results of multiple linear regression analysis performed to assess the independent relationship between DNA level, the study (ours vs. Bi et al. 2008) and gestational age.

Variables	DNA level			
	β	Robust SE	95% CI β	P-value
Study (ours vs Bi et al. 2008)	−103	14.4	−131; −74	0.0001
Gestational age	20.2	2.75	14.7; 25.6	0.0001
Intercept	−276	43.3	−362; −190	0.0001

The multiple linear regression analysis model adjusted for gestational age (R^2^ = 0.42).

For the subgroup of the 41 uncultured samples of amniocytes of less than 16 weeks of gestation with or without chromosomal abnormalities, between 28.3 and 238.7 ng of DNA per 1 ml of AF were extracted (Table 3). The quantities of DNA extracted from the 41 samples of uncultured amniotic fluid were greater than those using the protocol described by Bi et al. [8] (98.1 ng vs. 36.4 ng, respectively; p = 0.0003 See Table 4). The extraction protocol was reproducible and allowed us to obtain sufficient amounts of DNA with an average A260/A280 ratio of 1.92 for the 41 samples. The average A260/A230 ratio was dependent on the gestational age (0.83<16 wg, 1.53 17–20 wg, 1.73 21–22 wg and 1.66>23 wg). The average A260/A280 ratio for the ninety samples was 1.82, indicating that the DNA was of good quality. High-quality genomic DNA samples should have an A260/A280 ratio of 1.8 to 2.0, indicating the absence of contaminating proteins. The use of Rnase in 9 cases did not show any difference for the DO260/280 with or without Rnase (p = 0.699) (Table S1 and Table S2).

### Array-CGH

#### Ten samples <16 weeks of gestation

**Figure 1:**
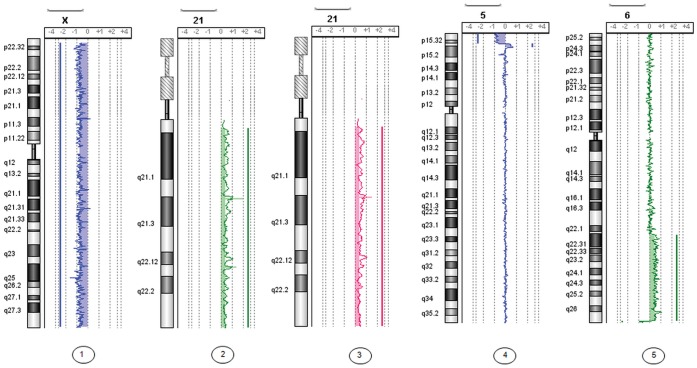
Genome views of five number and structural abnormalities characterized by array-CGH, extracted from 1 ml of uncultured amniotic fluid from patients at less than 16 weeks of gestation. (cases 1 to 5).

Sensitivity: Chromosome imbalances in the five samples of uncultured amniotic fluid of less than 16 weeks of gestation analyzed by array-CGH 4*44 K Agilent were detected and characterized (Table 1). Three number abnormalities and two structural abnormalities (Figure 1 and Table 1) were characterized in DNA from the uncultured amniotic fluid. The quality control from Agilent with DLRS (Derivative Log Ratio Spread) showed from excellent to good quality for the five samples (DLRS between 0.09 to 0.31, Table 1). The smallest amount of DNA with abnormalities available for array-CGH experiments was 50 ng/ml (case 3). Only one case (case 6) showed a slight discordance in favor of the array-CGH, which detected a 2.3 Mb 5p15 duplication not identified by prenatal karyotyping in addition to the previously detected imbalance.

Specificity: The five samples with normal karyotypes showed that the smaller the amount of DNA, the greater the DLRS (between 0.26 and 0.60). Detection of CNV showed an average of three per patient ranging from 400 bp to 600 kb; all were found in the Database of Genomic Variants (DGV).

#### Twelve samples >16 weeks of gestation

**Figure 2:**
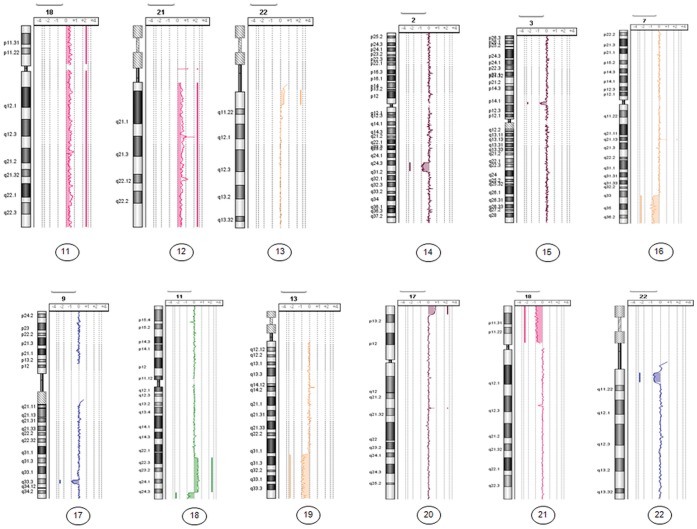
Genome views of 12 chromosomal abnormalities characterized by array-CGH, extracted from 1 ml of uncultured amniotic fluid from patients at more than 16 weeks of gestation. (cases 11 to 22).

For the 12 samples of uncultured amniotic fluid with aneuploidy or structural abnormalities at more than 16 weeks’ gestation, all of the chromosomal imbalances were detected and characterized by array-CGH (Table 2 and Figure 2).

## Discussion

In recent years, there has been considerable progress in the use of array-CGH in the postnatal diagnosis of mental retardation [21]. Studies of fetal tissue with multiple malformations or spontaneous miscarriages revealed 10% of unbalanced rearrangements [22]–[26]. Larrabee et al. [1] were the first to test the array-CGH technique to analyze fetal DNA extracted from AF cffDNA and to compare the results obtained by array-CGH with those obtained with conventional karyotyping. In the literature, in a prenatal context, array-CGH analysis of cultured AF has been applied to marker or apparently balanced translocations, and has allowed genetic counseling [27], [28].

Five retrospective [1], [2], [5], [11], [29] and twelve prospective studies [3], [4], [6], [7], [8], [9], [11], [12], [13], [14], [15], [16] of prenatal array-CGH with different resolutions and involving uncultured amniotic fluid have been published (Table 6 and 7). Only one of them matched the aims of the present study, i.e. to describe a new method for DNA extraction from small quantities of AF, but the authors only tested two samples <16 WG [8]. Our results suggested that our improved protocol gave significantly higher quantities of DNA than did the Bi et al. [8] protocol (Table 4 and 5). Our new protocol for DNA extraction from uncultured AF generated DNA of superior quality in greater quantities for array-CGH from less AF. This protocol allowed the rapid analysis of the fetal genome without the need for cell culture, and therefore earlier diagnosis, or retrospective studies when only a small quantity of frozen AF is available. Even if the DLRS are not-optimal according to the recommendation protocols, all the known chromosomal rearrangements were confirmed. In one case, the array-CGH also showed a duplication (5p15, case 4) that was not detected by prenatal karyotyping because the size of the abnormality was too small to be detected by karyotype. Therefore, this method should be restricted to certain cases when biologists only have a small quantity of sample available. For array-CGH analysis on an Agilent platform, a yield of 50 ng is sufficient to obtain results using the protocol described here, without background noise. Many post-natal reports have demonstrated the sensitivity, specificity and accuracy of array-CGH in detecting large and small imbalances. In our prenatal study, array-CGH showed reproducible results with high sensitivity and specificity in the analyses of 10 samples of uncultured amniotic fluid samples of less than 16 weeks of gestation. In our retrospective study, we were able to detect and characterize all of the rearrangements using 1 ml of uncultured AF before or after 16 weeks of gestation. We tested the DNA extraction protocol described by Rickman et al. [2] with 1 ml of uncultured AF without success (the amount of DNA extracted was between 33 and 130 ng from ten samples of uncultured amniotic fluid, gestational age ranging between 19 and 24 weeks). However, in prenatal studies, array-CGH is limited by the fact that this technique is unable to detect polyploidy and balanced chromosomal rearrangements. Furthermore, array-CGH has the potential to uncover unwanted information in the prenatal setting (genes that predispose for cancer, heterozygoty for autosomal recessive disease, carrier of X linked disease), which creates ethical problems in a prenatal context. In prenatal diagnosis, the detection of new CNV not reported in the Database of Genomic Variants (DGV), or variants with incomplete penetrance without ultrasound abnormalities could lead to major difficulties in genetic counseling.

**Table 6:** Retrospective study of amniotic fluid (AF) by array-CGH.

Study	Number of AF	Concordance	Array types	DNA preparation	Volume of AF
Larrabee et al. [2004]	21	21/21	BAC 300 clones	AF cffDNA	10 ml
Rikman et al. [2006]	30	29/30	BAC 600 clones	Uncultured AF	1 ml
Choe et al. [2007]	15	15/15	BAC 1440 clones	Uncultured AF with amplification	4 ml
Lapaire et al. [2007]	10	9/10	BAC 434 clones	AF cffDNA	10 ml
Gruchy et al. [2011]	38	–	BAC 3200 clones	AF cffDNA, cultured AF	10 ml

AF cffDNA, amniotic fluid supernatant cell-free fetal DNA.

**Table 7:** Prospective study of amniotic fluid (AF) by array-CGH.

Study	Number of AF	Indication	Abnormalities detected by array-CGH %	Abnormalities not detected on karyotype %	Array types	DNA preparation	Volume of AF
Sahoo et al. [2006]	56	AMA, AU	5	0	BAC 1476 clones	Uncultured AF with amplification (n = 26) Cultured AF (n = 30)	5 ml
Miura et al. [2006]	13	AU	46	0	BAC 50 clones	AF cffDNA	10 ml
Choe et al. [2007]	118	AMA, AU	5	0	BAC 1440 clones	Uncultured AF with amplification	4 ml
Van den Veyver et al. [2008]	254	AMA, AU	5	2	BAC 1476 clones	Uncultured AF with (n = 50) or without (n = 38) amplification	5–7 ml
						Cultured AF (n = 166)	
Shaffer et al. [2008]	151	AMA, AU	1.3	1.3	BAC 4670 clones	Cultured AF (n = 151)	no data
Bi et al. [2008]	15	AMA, AU	6.6	0	Oligo 44 000	Uncultured AF with (n = 2) or without (n = 13) amplification	5–10 ml
Kleeman et al. [2009]	50	AU	2	2	BAC 1887 clones	Uncultured AF (n = 6)	5–48 ml
					BAC 4685 clones	Cultured AF (n = 44)	
Coppinger et al. [2009]	188	AMA, AU	3.8	2.7	BAC 4670 clones	Uncultured AF (n = 30)	no data
					Oligo 105 000	Cultured AF (n = 158)	
Maya et al. [2010]	204	AMA, AU	2.6	1.1	BAC 1476 clones	Uncultured AF (n = 123)	no data
					Oligo 105 000	Cultured AF (n = 81)	
Park et al. [2011]	4033	AMA, AU	1.8	0.27	BAC 1440 clones	Uncultured AF with amplification	4 ml
Fiorentino et al. [2011]	938	AMA, AU	3.3	0.9	BAC 3161	Uncultured AF (n = 924)	5–15 ml
						Cultured AF (n = 15)	
Lee et al. [2012]	2996	AMA, AU	1.2	1.1	BAC 1093 clones	Uncultured AF (n = 19)	15 ml
					Oligo 60 000	Cultured AF (n = 2977)	
					Oligo 105 000		

AMA, advanced maternal age; AU,abnormal ultrasound.

In conclusion, this study describes a reproducible method for DNA extraction from only 1 ml of uncultured amniotic fluid with significantly greater quantities recovered in our study than in the Bi et al. study [8]. This improved protocol for DNA extraction from 1 ml of uncultured AF permits array-CGH analysis in prenatal diagnosis from patients at less than 16 weeks’ gestation. This technique can be applied to retrospective and prospective studies and benefits from a number of advantages: short turn-around time, absence of culture and high resolution. Until array-CGH becomes a standard technique for prenatal diagnosis, it should be considered a complement to the standard prenatal karyotyping.

## Supporting Information

Table S1 **Summary for variables (do260280) by categories of Rnase.**
(DOC)

Table S2 **Linear regression for variables (do260280) by categories of Rnase.**
(DOC)
