# Supplementary material for: An Improved Method to Extract DNA from 1 ml of Uncultured Amniotic Fluid from Patients at Less than 16 Weeks’ Gestation
Source: PLoS One. 2013 Apr 2;8(4):e59956. doi: 10.1371/journal.pone.0059956 (PMC3614959; doi:10.1371/journal.pone.0059956)
Supplement: Supplementary file 1 [file pone.0059956.s001.doc]

Supplemental Table S1: Summary for variables (do260280) by categories of rnase

rnase	N	mean	sd	min	max	p50	p25	p75
0	32	1.94125	.361384	1.4	2.66	1.865	1.66	2.19
1	9	1.86	.6156703	1.19	3.34	1.78	1.5	1.97
Total	41	1.923415	.4221173	1.19	3.34	1.82	1.66	2.1
