# Supplementary material for: An Improved Method to Extract DNA from 1 ml of Uncultured Amniotic Fluid from Patients at Less than 16 Weeks’ Gestation
Source: PLoS One. 2013 Apr 2;8(4):e59956. doi: 10.1371/journal.pone.0059956 (PMC3614959; doi:10.1371/journal.pone.0059956)
Supplement: Supplementary file 2 [file pone.0059956.s002.doc]

Supplemental Table S2: Linear regression (Number of obs = 41; F(1, 39) = 0.15 Prob > F = 0.6990; R-squared = 0.0065; Root MSE = .4261)

do260280	Coef.	Robust Std. Err.	t	P>|t|	[95% Conf.	Interval]
rnase	-.08125	.2085984	-0.39	**0.699**	-.5031801	.3406801
_cons	1.94125	.0644703	30.11	0.000	1.810847	2.071653
